# The Salicylates and Hæmorrhages in Enteric Fever

**Published:** 1883-05

**Authors:** 


THE SALICYLATES AND HÆMORRHAGES IN ENTERIC FEVER.—Dr. James Fergusson, of Perth, writes: "At the time when salicylic acid and its compounds are receiving so much attention, may the following facts be regarded as at least worthy of statement? Last year, while resident in the infirmary here, I had an opportunity of testing the efficacy of certain drugs as antipyretics in enteric fever. These agents were used successively, each over a group of cases, and included the salicylate of soda. The latter had not been long in use when an increased frequency of hæmorrhages from the bowel raised the question, Could the salicylate be favoring the production of that complication of the malady? Whether it were or not, the suspicion aroused, dictated the withdrawal of the salt from use in cases of typhoid. Shortly afterward, I noticed that a foreign observer had reported the salicylate of bismuth, and, I think, also salicylate acid (though of the latter I cannot be certain, as I am not able now to find the report in question), to cause intestinal and nasal hæmorrhages. The subject would not have been revived by me at present, but for the recent experience of my successor in the resident's office at the above-mentioned institution, D. H. McLean Wilson, who joins me in placing the facts before the public. Dr. Wilson, in having recourse to the soda-salt in typhoid, found the same striking frequency of hæmorrhage to follow closely. His employment of the agent differed from mine, in that he administered the small doses of ten to fifteen grains frequently over the twenty-four hours, while I gave half-drachm or drachm doses at longer intervals apart. In the other respect, however, our experiences have been so similar as to warrant the fact being brought under notice, so that the important practical question involved, may, if possible, be decided by the evidence of a number of observers."—*British Medical Journal.*

